# One-pot preparation of nanoporous Ag-Cu@Ag core-shell alloy with enhanced oxidative stability and robust antibacterial activity

**DOI:** 10.1038/s41598-017-10630-5

**Published:** 2017-08-31

**Authors:** Xue Liu, Jing Du, Yang Shao, Shao-Fan Zhao, Ke-Fu Yao

**Affiliations:** 1 School of Materials Science and Engineering, Tsinghua University, Beijing, 100084 People’s Republic of China; 2 Institute of Materials, China Academy of Engineering Physics, Mianyang, 621900 People’s Republic of China; 3 Institute of Biomechanics and Medical Engineering, School of Aerospace, Tsinghua University, Beijing, 100084 People’s Republic of China; 4 Qian Xuesen Laboratory of Space Technology, Beijing, 100094 People’s Republic of China

## Abstract

Metallic core–shell nanostructures have inspired prominent research interests due to their better performances in catalytic, optical, electric, and magnetic applications as well as the less cost of noble metal than monometallic nanostructures, but limited by the complicated and expensive synthesis approaches. Development of one-pot and inexpensive method for metallic core–shell nanostructures’ synthesis is therefore of great significance. A novel Cu network supported nanoporous Ag-Cu alloy with an Ag shell and an Ag-Cu core was successfully synthesized by one-pot chemical dealloying of Zr-Cu-Ag-Al-O amorphous/crystalline composite, which provides a new way to prepare metallic core–shell nanostructures by a simple method. The prepared nanoporous Ag-Cu@Ag core-shell alloy demonstrates excellent air-stability at room temperature and enhanced oxidative stability even compared with other reported Cu@Ag core-shell micro-particles. In addition, the nanoporous Ag-Cu@Ag core-shell alloy also possesses robust antibacterial activity against *E. Coli* DH5α. The simple and low-cost synthesis method as well as the excellent oxidative stability promises the nanoporous Ag-Cu@Ag core-shell alloy potentially wide applications.

## Introduction

Metallic nanostructures possess many novel properties that are profoundly different from their bulk counterparts, which endow them wide applications in sensors, catalyst and energy^1–5^. As we have known that the performance of the materials with nanostructure is heavily depended on their constituents arrangement^6^, metallic core–shell nanostructures with less cost of noble metal, designed by compositing different metallic phases together, have shown better catalytic, optical, electric, and magnetic properties than monometallic nanostructures^6–9^. The most widely used preparation techniques of core–shell nanostructures, such as microemulsion method, epitaxial growth, microwave synthesis, electrochemical dealloying and multi-step reduction method, are limited by the complicated procedures, high costs, or difficulties to extend to large scales^1, 10–14^. Although metallic materials with core-shell nanostructures have demonstrated many potential applications, there are still many difficulties to obtain such core–shell nanostructured materials in a simple and less costly way towards routine large-scale synthesis^15, 16^. Thus the development of simple and inexpensive metallic core–shell nanostructures preparation method is of great importance.

Nanostructured copper and silver are widely used in the electronic devices, sensors, catalyst and anti-bacteria materials. But the spontaneous oxidation of copper and the relatively expensive price of silver apparently limited their applications^7, 17–20^. The emergence of Ag-Cu core–shell alloy with a silver shell is expected to solve this predicament^6, 7, 9^. However, the simple and inexpensive method to obtain Ag-Cu core–shell nanostructured materials still remains challenging. In present work, through introducing a proper amount of oxygen into the Zr-Cu-Ag-Al alloy, Cu network supported nanoporous Ag-Cu@Ag core-shell alloy was successfully prepared by a simple one-pot chemical dealloying process at room temperature. The nanostructure morphology evolution as well as the anti-oxidation and antibacterial properties of nanoporous Ag-Cu@Ag core-shell alloy were also investigated. By using this method, other functional materials with core-shell nanostructure may be easily synthesised, which can promote their potential applications.

## Results

### Characterization of the Cu network supported nanoporous Ag-Cu@Ag core-shell alloy

**Figure 1:**
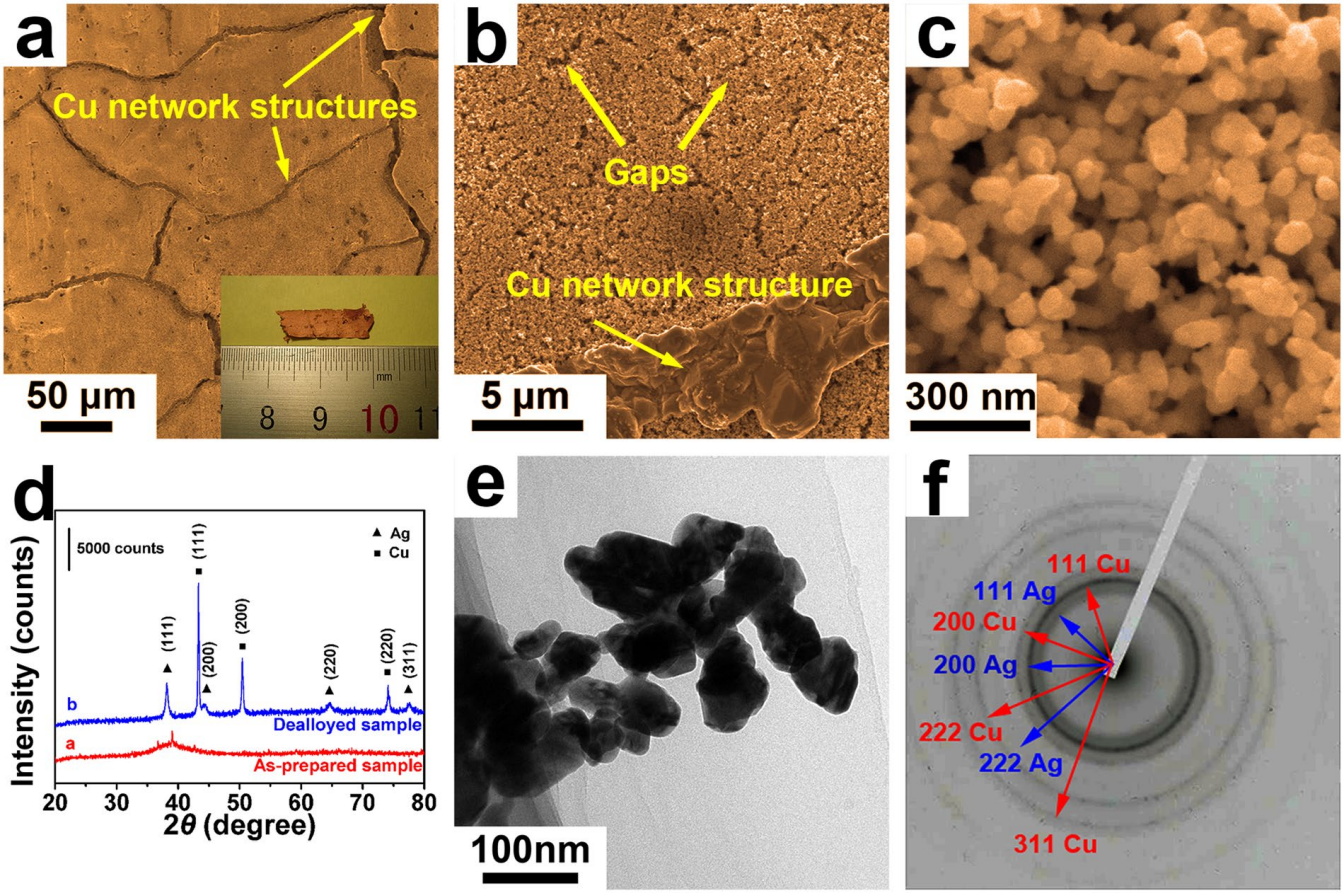
Morphology of the nanoporous Ag-Cu@Ag core-shell alloy. (**a**–**c**) SEM images of the 24 h dealloyed sample with different magnifications. Inset in (**a**) is the optical photograph of the 24 h dealloyed Zr-Cu-Ag-Al-O ribbon sample. (**d**) XRD spectra of the as-prepared (line a) and the 24 h dealloyed (line b) Zr-Cu-Ag-Al-O ribbon samples. (**e**) Bright field TEM image of the 24 h dealloyed Zr-Cu-Ag-Al-O ribbon sample. (**f**) The SAED pattern of (**e**).

The true composition of the Zr-Cu-Ag-Al-O ribbon sample was confirmed to be about Zr_38_Cu_40_Ag_7_Al_7_O_8_ by energy dispersive spectrometer (EDS) in scanning electron microscope (SEM). A hydrofluoric acid solution of 0.05 M was used to partially remove t﻿he﻿ metallic elements from the Zr-Cu-Ag-Al-O ribbon, which is termed as a dealloying process. When the dealloying process began, gas bubbles started to emerge on the surface of the sample, and the colour of the sample gradually turned into brassy yellow. After 24 h dealloyed, there were no more gas bubbles generated on the sample surface and the colour of the sample didn’t change any more. The dealloying process was believed to be finished. The optical photograph of the 24 h dealloyed Zr-Cu-Ag-Al-O ribbon sample with a dimension of ~6 mm × 17 mm × 25 μm is shown in the inset of Fig. 1a, where the sample appears brassy yellow in colour. The 24 h dealloyed Zr-Cu-Ag-Al-O ribbon sample is quite brittle, but can be carefully handled with tweezers. Figure 1a shows the SEM image of the 24 h dealloyed Zr-Cu-Ag-Al-O ribbon sample. It was noticed that the sample is composed of grain-like areas with feature sizes of ~100 μm, and network structures (marked by arrows) can be found between the grain-like areas. The high-magnification SEM image of the network structures is shown in Fig. 1b. It was found that the network structure is assembled by many blocks, and the width of the network structure was measured to be ~4 μm. With the help of EDS in SEM, the network structure was confirmed to be constituted by pure copper. From Fig. 1b, it can be also found that the grain-like area is of nanoporous structure. Some gaps were noticed on the nanoporous structure (seeing Fig. 1b), which may be associated with the grain boundaries in the as-prepared Zr-Cu-Ag-Al-O ribbon. Figure 1c shows the high-magnification SEM image of the nanoporous structure, where the diameters of the ligament (structure connecting the adjoining nanopores) were measured to be ~60 nm. Two kinds of nanoporous structures with different pore sizes can be observed. The diameters of the small nanopores and large nanopores were measured to be ~15 nm and ~40 nm, respectively. With the help of EDS in SEM, the composition of the nanoporous structure was confirmed to be Cu_44_Ag_56_. Supplementary Fig. S1 shows the corresponding EDS plot, where no obvious oxygen peak was found.

Figure 1d presents the X-ray diffraction (XRD) spectra of the as-prepared and the 24 h dealloyed Zr-Cu-Ag-Al-O ribbon samples. There are a few weak diffraction peaks on a diffuse scattering peak in the XRD spectrum of the as-prepared Zr-Cu-Ag-Al-O ribbon sample, indicating that some crystalline phases formed inside the amorphous matrix. Those crystalline phases were identified to be Cu_2_O, ZrO and ZrCu, as shown in Supplementary Fig. S2. Since the as-prepared Zr_48_Cu_36_Ag_8_Al_8_ ribbon sample without oxygen addition is of fully glassy state (as shown in Supplementary Fig. S3) and the glass forming ability of Zr-based metallic glasses (MGs) is very sensitive to oxygen^21, 22^, the partial crystallization of the Zr-Cu-Ag-Al-O ribbon should be caused by oxidation. In the XRD spectrum of the 24 h dealloyed Zr-Cu-Ag-Al-O ribbon sample, only sharp peaks corresponding to face centered cubic (FCC) Cu and FCC Ag phases but no other intermetallic phase were noticed. The results show that the dealloyed Zr-Cu-Ag-Al-O ribbon sample is of nanoporous Ag-Cu alloy with separated Ag and Cu phases.

Transmission electron microscope (TEM) was employed to figure out the phase distribution in the nanoporous Ag-Cu alloy. Figure 1e is the bright field TEM image of the nanoporous Ag-Cu alloy. Two kinds of nanopores with diameters of ~15 nm and ~40 nm, and ligaments with a smooth surface and an average diameter of ~60 nm could be noticed, which are consistent with the SEM results in Fig. 1c. The selected area electron diffraction (SAED) pattern on the nanoporous Ag-Cu alloy in Fig. 1e is shown in Fig. 1f, where diffraction rings were indexed as FCC Ag and FCC Cu phases. Those results confirm that the nanoporous structure is constituted by FCC Ag and FCC Cu phases. The continuous diffraction rings in the SAED pattern also indicate the fine-grain structure of the nanoporous Ag-Cu alloy.

**Figure 2:**
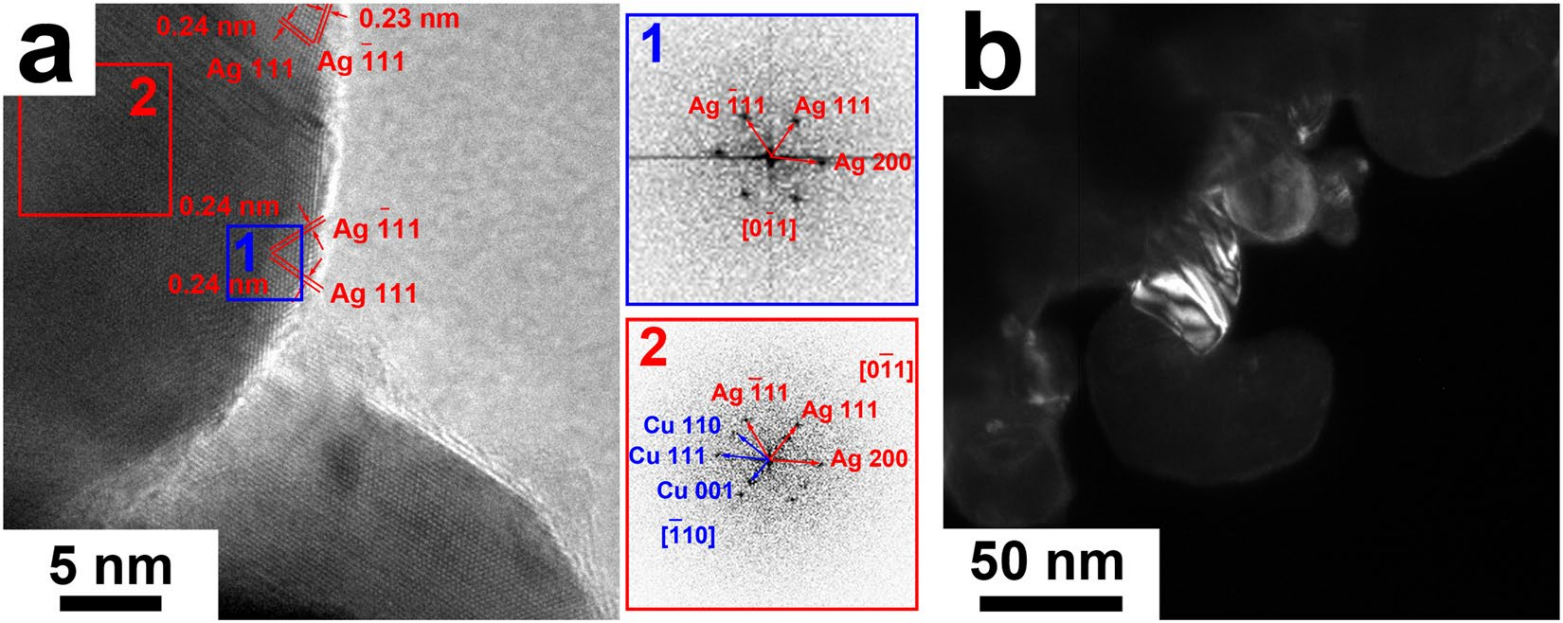
TEM images of the nanoporous Ag-Cu@Ag core-shell alloy. (**a**) The HRTEM image of the nanoporous Ag-Cu@Ag core-shell alloy with its selected-area FFT results in the inserts. (**b**) The dark field TEM image corresponding to the diffraction spots of the {111} plane of Ag (marked by red circle in Fig. S5b).

Figure 2a is a typical high-resolution transmission electron microscope (HRTEM) image of the nanoporous Ag-Cu alloy. No detectable oxide layers can be found on the surface. On the edge of almost all the nanoporous Ag-Cu alloy, the interplanar spacing is measured to be 2.3–2.4 Å, which is corresponding to the interspacing of the {111} planes of Ag. Similar results were noticed in all other nanoporous Ag-Cu samples (seeing Supplementary Fig. S4). The inserts 1 and 2 in Fig. 2a show the fast Fourier transform (FFT) results of the surface area 1 and core area 2 marked in Fig. 2a, respectively. The contrast of the FFT results has been adjusted to make the patterns clear. Through careful indexing and analysis of the obtained FFT results, it has been confirmed that the surface layer of the nanoporous Ag-Cu alloy is made of pure FCC Ag grains, while the core area is made of FCC Ag and FCC Cu grains. Thus, the nanoporous Ag-Cu alloy is of core-shell structure with an Ag shell and an Ag-Cu core. Supplementary Fig. S5 shows another bright field TEM image of the nanoporous Ag-Cu alloy and its corresponding diffraction pattern with dispersed spots. Figure 2b shows the dark field TEM image corresponding to the diffraction spots that were indexed as the {111} plane of Ag (marked by a red circle in Supplementary Fig. S5b). By comparing Fig. 2b with the bright field TEM image shown in Supplementary Fig. S5a, it can be found that the surface area is brighter than most of the core areas, indicating that the nanoporous Ag-Cu alloy is coated with an Ag shell. The above results declare that Cu network supported nanoporous Ag-Cu@Ag core-shell alloy has been successfully synthesised by employing the simple one-pot chemical dealloying technique.

### Formation mechanism of the Cu network supported nanoporous Ag-Cu@Ag core-shell alloy

To figure out the formation mechanism of the Cu network supported nanoporous Ag-Cu@Ag core-shell alloy, the Zr_48_Cu_36_Ag_8_Al_8_ MG ribbon without oxygen addition was dealloyed under the same condition, but only Ag-Cu nanoporous structure with diffusely distributed Ag and Cu was obtained^23^. Therefore, the formation of the Cu network supported nanoporous Ag-Cu@Ag core-shell alloy may be closely related to the oxygen addition.

**Figure 3:**
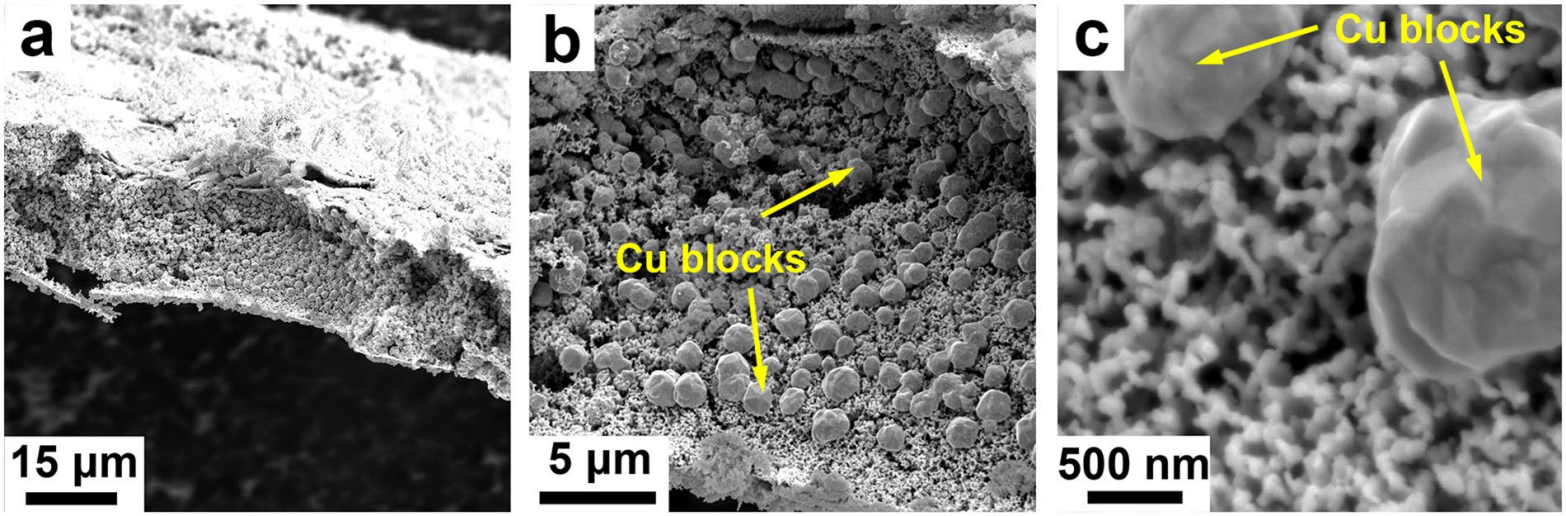
SEM images of the dealloyed Zr_48_Cu_36_Ag_8_Al_8_ MG ribbon that was subjected to oxidation in the air at 473 K for 30 min with different magnifications.

To confirm this suggestion, the Zr_48_Cu_36_Ag_8_Al_8_ MG ribbon was subjected to oxidation in the air at 473 K for 30 min, and then dealloyed under the same condition as the Zr-Cu-Ag-Al-O ribbon. After dealloying, several layers were formed on the dealloyed sample, as shown in Fig. 3a. The outer layer was confirmed to be ZrO_2_, then followed by a layer of CuO and Cu_2_O and a middle layer of Cu_56_Ag_44_ alloy, as shown in Supplementary Fig. S6. Figure 3b and c show the SEM images of the Cu_56_Ag_44_ middle layer, which is also constituted by Cu blocks and nanoporous Ag-Cu@Ag core-shell alloy. But the Cu blocks are dispersedly distributed, and no Cu network structures are formed. This phenomenon is caused by the structure difference between the oxidized amorphous Zr_48_Cu_36_Ag_8_Al_8_ ribbon and the partially crystallized Zr-Cu-Ag-Al-O ribbon. In the partially crystallized Zr-Cu-Ag-Al-O ribbon, the Cu mainly precipitate at the grain boundaries, while there is no grain boundary in the oxidized amorphous Zr_48_Cu_36_Ag_8_Al_8_ ribbon, making the Cu precipitate dispersedly. By comparing this result with the dealloying result of the unoxidized Zr_48_Cu_36_Ag_8_Al_8_ MG ribbon, it can be confirmed that the formation of the Cu network supported nanoporous Ag-Cu@Ag core-shell alloy is indeed closely related to the addition of oxygen.

The structures and compositions of Zr-Cu-Ag-Al-O ribbon samples dealloyed for different time were also investigated. The SEM images of two typical samples dealloyed for 10 min and 1 h are shown in Supplementary Fig. S7a and b, respectively. Some annular wrinkles were noticed on the surfaces, which may be related to grain boundaries. With extending dealloying time, more annular wrinkles were formed, and the sample surface was finally bestrewed by the annular wrinkles. In the high-magnification images of the samples dealloyed for 10 min and 1 h, uniform nanopores structures were found, and the nanopores were measured to be ~10 nm and ~20 nm, respectively. It can be easily found that the porosity and the nanoporous diameter increase with extending dealloying time.

**Figure 4:**
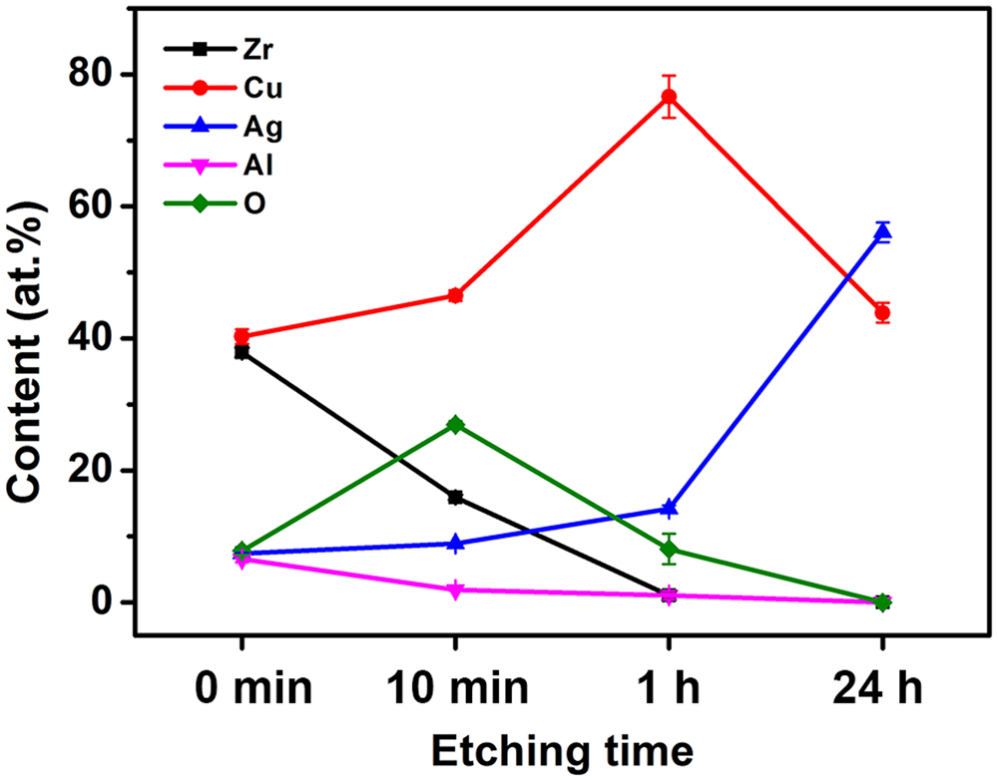
The composition variation of the Zr, Cu, Ag, Al and O during the dealloying process.

The compositions of the dealloyed Zr-Cu-Ag-Al-O ribbon sample at different dealloying time were collected and investigated by EDS. The composition results are presented in Fig. 4, and the data are listed in Supplementary Table S1. The content of Zr and Al gradually reduces to zero during the dealloying process, and that of Ag and Cu keeps increasing until Zr and Al are etched off. While the oxygen content rapidly rises in the first 10 min and then drops. The quick rise of oxygen should be related to the rapid oxidation of the newly formed nanostructures, which caused by the dissolved oxygen in the hydrofluoric acid solution. It was noticed that the atom ratio of Cu/Ag keeps around 5:1 before Zr and Al are etched off. Since the electrode potential of Cu is significantly different from that of Ag, the dissolution ratio would be different under the same corrosion condition. Therefore during the first hour of the dealloying process, the etching of Zr and Al and their oxide is the main reaction. After the first hour, both the copper content and the oxygen content drop while the silver content rises, indicating the dissolution of copper oxide.

**Figure 5:**
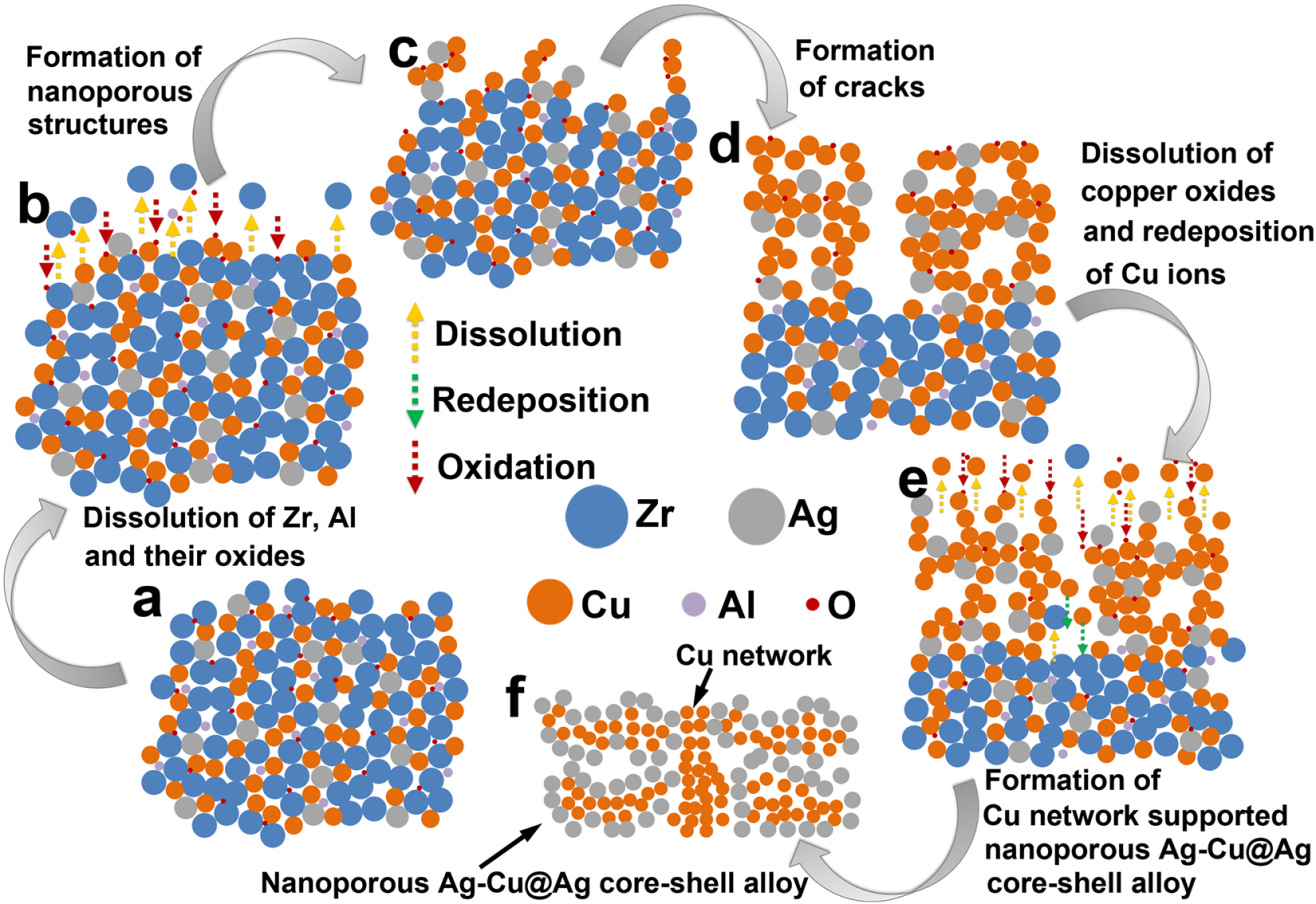
Illustration of the evolution of the nanoporous Ag-Cu@Ag core-shell alloy. (**a**) The as-prepared Zr-Cu-Ag-Al-O ribbon. (**b**) The dissolution of Zr, Al and their oxides. (**c**) The formation of nanoporous structures with high Cu and Ag content. (**d**) The cracks form in the nanoporous structures when the dealloying depth reaches a critical value. (**e**) The dissolution of Zr, Al and the copper oxides, and the redeposition of Cu ions. (**f**) The formation of Cu network supported nanoporous Ag-Cu@Ag core-shell alloy.

Figure 5 illustrates the possible evolution of the Cu network supported nanoporous Ag-Cu@Ag core-shell alloy. Before dealloying, the sample is composed of amorphous phases and crystalline phases with some oxides, such as Cu_2_O and ZrO, as shown in Fig. 5a. When the dealloying process starts, Zr, Al and the oxides are preferentially corroded out due to their relatively higher potentials than those of Cu and Ag, leading to the exposing of Cu and Ag atoms. At the same time, the newly exposed Cu, Zr and Al are oxidized by the dissolved oxygen in the solution, resulting in the rise of oxygen content in the sample, as presented in Fig. 5b. Just like the dealoying process in Au-Ag system^24^, the exposed Cu and Ag atoms subsequently diffuse around and start to agglomerate into islands, and finally resulting in the formation of nanoporous structures with more Cu and Ag content in Fig. 5c. With the extending of dealloying time, the porosity would increase due to the continuous dissolution of Zr and Al atoms, and the nanopore size would increase due to the diffusing of Cu and Ag atoms. When the dealloying depth reaches a certain value, cracks form due to the faster corrosion rate in the grain boundaries and the corrosion stress, as shown in Fig. 5d. Then the Cu oxides on the sample surface are dissolved into the solution, and Cu ions are redeposited on the cracks under the reduction of Zr and Al atoms, as presented in Fig. 5e. During this process, Ag atoms are enriched on the sample surface, and Cu atoms are gathered on the cracks to form the Cu network. Finally, all the Zr, Al and oxides are dissolved, and the Ag atoms occupy the whole surface. Then the dealloying process ends, and the Cu network supported nanoporous Ag-Cu@Ag core-shell alloy are formed, as displayed in Fig. 5f. Since the dealloying process will end when the Ag atoms occupy the whole surface, the thickness of the Ag shell in the nanoporous Ag-Cu@Ag core-shell alloy may be quite stable.

### Morphology tailoring of the Cu network supported nanoporous Ag-Cu@Ag core-shell alloy

It is well known that the pore size of the dealloyed porous materials can be controlled by adjusting the solution concentration^25^. Generally, with the increasing concentration of the dealloying solution, the diameter of the pore as well as the ligament increases^25^. Then it is possible to tailor the morphology of the Cu network supported nanoporous Ag-Cu@Ag core-shell alloy by dealloying the Zr-Cu-Ag-Al-O ribbon in hydrofluoric acid with different concentrations.

**Figure 6:**
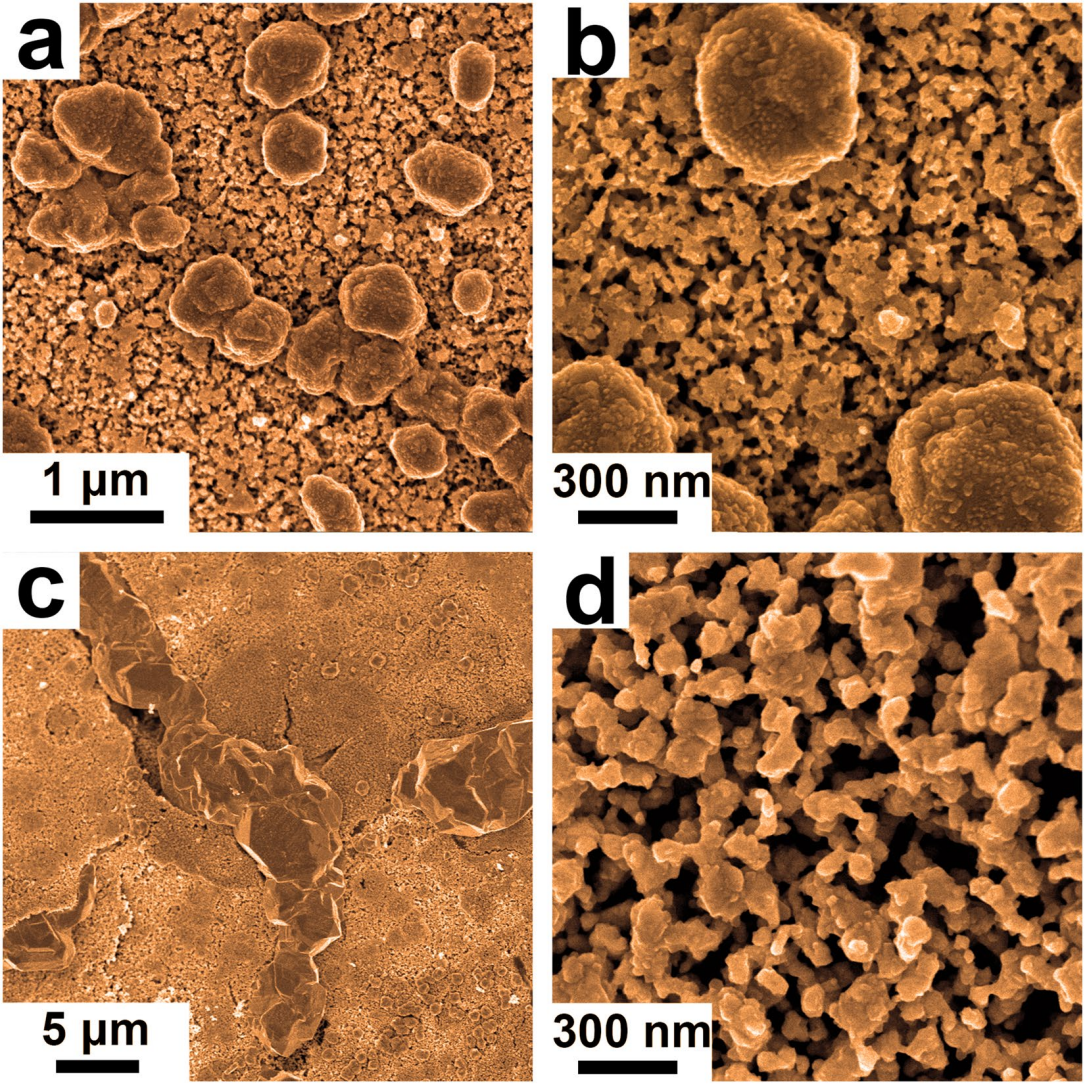
SEM images of the 24 h dealloyed Zr-Cu-Ag-Al-O ribbon in hydrofluoric acid with different concentrations. (**a**) Zr-Cu-Ag-Al-O ribbon dealloyed in 0.01 M hydrofluoric acid for 24 h. (**b**) The enlarged SEM image of (**a**). (**c**) Zr-Cu-Ag-Al-O ribbon dealloyed in 0.25 M hydrofluoric acid for 24 h. (**d**) The enlarged SEM image of (**c**).

Figure 6a shows the SEM image of the Zr-Cu-Ag-Al-O ribbon dealloyed in 0.01 M hydrofluoric acid for 24 h. The dealloyed sample is also composed with Cu and nanoporous Ag-Cu@Ag core-shell alloy. Different from the sample dealloyed in 0.05 M hydrofluoric acid (seeing Fig. 1b), the Cu forms blocks but not network structure. The diameter of the Cu block is ~500 nm, which is much smaller than the width of the Cu network presented in Fig. 1b. Figure 6b presents the enlarged SEM image of Fig. 6a. Some cracks were noticed in the nanoporous Ag-Cu@Ag core-shell alloy. The diameters of the pores and the ligaments were measured to be ~30 nm and ~20 nm, respectively. After dealloying in 0.25 M hydrofluoric acid, Cu network supported nanoporous Ag-Cu@Ag core-shell alloy was also prepared, as shown in Fig. 6c. The width of the Cu network is ~6 μm. The enlarged SEM image of the nanoporous Ag-Cu@Ag core-shell alloy is shown in Fig. 6d, where the diameters of the pores and the ligaments were measured to be ~100 nm and ~60 nm, respectively. Therefore, with increasing dealloying solution’s concentration, the size of the Cu structure increases, and the diameters of the pores as well as the ligaments in the nanoporous Ag-Cu@Ag core-shell alloy also increase. The increasing of the hydrofluoric acid concentration can promote the etching rate of the Zr and Al, which will make the etching thickness increasing. The rapid etching of Zr and Al rapidly introduces many vacancies. But due to the limit of the diffusion rate, there isn’t enough time for the remained Ag and Cu atoms diffusion to form small particles. When the etching progresses far enough, the remained Ag and Cu atoms will collapse into larger particles. Then with the increasing hydrofluoric acid concentration, the diameter of the pore as well as the ligament increases. Therefore, by adjusting the concentration of the hydrofluoric acid, the morphology of the Cu network supported nanoporous Ag-Cu@Ag core-shell alloy can be tailored.

### Anti-oxygenic property of the Cu network nanoporous Ag-Cu@Ag core-shell alloy

**Figure 7:**
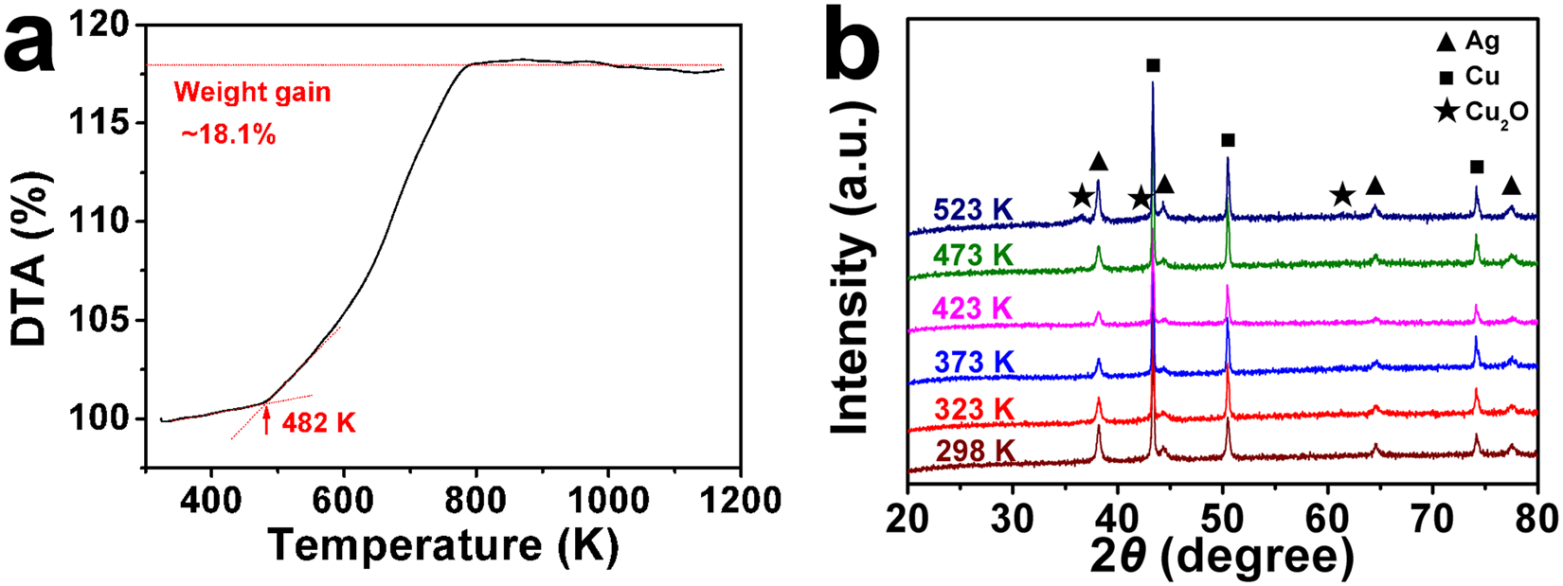
Anti-oxygenic property of the nanoporous Ag-Cu@Ag core-shell alloy. (**a**) TGA curve of the nanoporous Ag-Cu@Ag core-shell alloy with a heating rate of 10 K min^−1^. (**b**) XRD spectra of nanoporous Ag-Cu@Ag core-shell alloy oxidized at 298 K for 4 months, 323 K, 373 K, 423 K, 473 K and 523 K for 30 min in air condition.

The TGA plot of the nanoporous Ag-Cu@Ag core-shell alloy obtained in air is shown in Fig. 7. There is a slight decrease (less than 0.5%) on the TGA plot before 350 K, which comes from the loss of the absorbed water on the surface. Then the plot keeps rising before 795 K, which is corresponding to the oxidation of the nanoporous Ag-Cu@Ag core-shell alloy. There exists a slow oxidization stage and a quick oxidization stage separated by a critical temperature of 482 K on the TGA plot, indicating that the rapid oxidation of the nanoporous Ag-Cu@Ag core-shell alloy occurs at 482 K. Finally, the weight gain is saturated at ~18.1% after 795 K.

According to the TGA result, 298 K, 323 K, 373 K, 423 K, 473 K and 523 K were selected to be the oxidization temperatures to investigate the anti-oxidation properties of the Cu network supported nanoporous Ag-Cu@Ag core-shell alloy. After oxidization, XRD was used to evaluate the oxidation degree of the oxidized nanoporous Ag-Cu@Ag core-shell alloy, and the results were shown in Fig. 7b. Even after oxidized at 298 K for 4 months in ambient air, the XRD spectrum of the oxidized nanoporous Ag-Cu@Ag core-shell alloy is almost the same as that of the as-prepared sample, which shows the excellent air-stability of the nanoporous Ag-Cu@Ag core-shell alloy at room temperature. No obvious peaks correspond to oxides can be found in the spectra of the nanoporous Ag-Cu@Ag core-shell alloy oxidized at 323 K, 373 K, 423 K or 473 K for 30 min, while peaks corresponding to Cu_2_O emerged after oxidized at 523 K for 30 min. Thus, the obvious oxidation of the nanoporous Ag-Cu@Ag core-shell alloy occurs between 473 and 523 K, which consists with the rapid oxidation temperature (482 K) in the TGA result. Thus, the obvious oxidation of the Cu network supported nanoporous Ag-Cu@Ag core-shell alloy starts at 482 K. This temperature is much higher than that of our previously reported dendritic copper structures in sub-microscale^4^, which is also prepared by dealloying and possess an oxidation temperature of 449 K. What’s more, the obvious oxidation temperature of the prepared nanoporous Ag-Cu@Ag core-shell alloy is even higher than the reported core-shell Ag-Cu particles with 5 μm-diameter Cu core and 260 nm-thick Ag shell, which exhibit an obvious oxidation temperature below 473 K^19^.

### Antibacterial activity of the Cu network supported nanoporous Ag-Cu@Ag core-shell alloy

**Figure 8:**
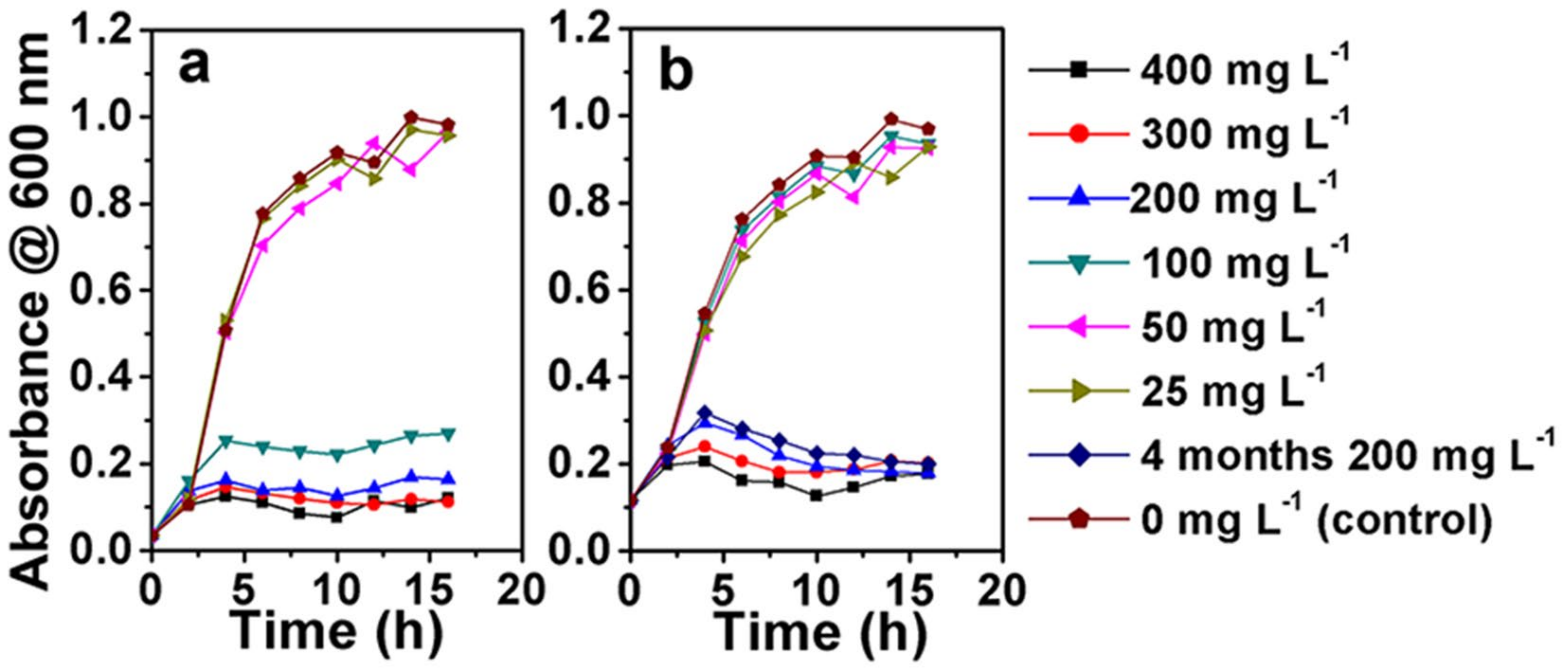
Antibacterial activity of the nanoporous Ag-Cu@Ag core-shell alloy. Representative batch growth profiles in the presence of nanoporous Ag-Cu@Ag core-shell alloy for initial concentrations of *E. Coli* DH5α: (**a**) 0.03 OD and (**b**) 0.11 OD.

Figure 8a shows the representative growth profiles in the presence of Cu network supported nanoporous Ag-Cu@Ag core-shell alloy for initial concentration of *E. Coli* DH5α of 0.03 optical density (OD) (~1.0 × 10^8^ colony-forming units (CFU) mL^−1^). The concentration of bacteria in the control group dramatically increases during the first 6 h, and then gradually stabilized around 1 OD (~3.5 × 10^9^ CFU mL^−1^). The addition of nanoporous Ag-Cu@Ag core-shell alloy below 50 mg L^−1^ has little influence on the batch growth profile of *E. Coli* DH5α. But the growth of the bacteria is significantly inhibited when the nanoporous Ag-Cu@Ag core-shell alloy addition exceeds 100 mg L^−1^, and the surviving bacteria population decreases with the increasing of nanoporous Ag-Cu@Ag core-shell alloy concentration. This result is consistent with the previous report that the concentration of Ag and Cu nanostructures has significant influence on their antibacterial activities^17, 20^. According to the previous work, the antibacterial activity of the Ag and Cu mainly comes from two aspects^17^. Firstly, the Ag and Cu ion can be adsorbed on the surface of the cytomembrane, which carries a net negative charge. Then the Ag and Cu ion can penetrate into bacteria and react with the sulfhydryl groups, finally resulting in cell death. Secondly, the Ag and Cu can produce free hydroxyl groups with the extending of oxygen, and the free hydroxyl groups can destroy the cell membranes causing deactivation of their activity. Besides that, the synergistic effect between the Cu and Ag can increase their antibacterial activity^17^, making the nanoporous Ag-Cu@Ag core-shell alloy exhibit excellent antibacterial activity.

It was noticed that even after 18 hours, no obvious degradation of antibacterial activity was observed. Since the prepared nanoporous Ag-Cu@Ag core-shell alloy possess excellent anti-oxygenic property, the long-term antibacterial effect is caused by their excellent stability.

Similar phenomenon was observed in the growth profile for initial concentration of *E. Coli* DH5α of 0.11 OD (~3.8 × 10^8^ CFU mL^−1^) (seeing Fig. 8b), but the critical effective concentration of nanoporous Ag-Cu@Ag core-shell alloy increased to 200 mg L^−1^. Compared with the growth profile for initial concentration of *E. Coli* DH5α of 0.03 OD, the surviving bacteria population is much larger under the same nanoporous Ag-Cu@Ag core-shell alloy concentration, which indicates that the concentration of the bacteria also has significant influence on the antibacterial activities of the nanoporous Ag-Cu@Ag core-shell alloy.

The antibacterial activities of the nanoporous Ag-Cu@Ag core-shell alloy that preserved in air for 4 months have also been investigated. It has been found that the bacteria growth profile with the nanoporous Ag-Cu@Ag core-shell alloy kept in air for 4 months is very similar to that with newly prepared one (shown in Fig. 8b), which indicating the robust antibacterial activity of the nanoporous Ag-Cu@Ag core-shell alloy. It is known that the oxidization of Cu and Ag nanostructures can cause the degradation of the antibacterial activity^17^. The present robust antibacterial activity of the Cu network supported nanoporous Ag-Cu@Ag core-shell alloy may arise from the excellent oxidative stability of the outermost Ag shell. As shown in Supplementary Fig. S8, repeated antibacterial activity tests were also conducted. The Cu network supported nanoporous Ag-Cu@Ag core-shell alloy exhibits very stable antibacterial activity.

Compared with the Ag or Cu nanoparticles, the nanoporous Ag-Cu@Ag core-shell alloy combines the excellent oxidative stability of Ag as well the low cost of Cu. Besides that, the nanoporous Ag-Cu@Ag core-shell alloy can exhibit better antibacterial activity due to the synergistic effect between the Cu and Ag. These make the Cu network supported nanoporous Ag-Cu@Ag core-shell alloy very attractive and promise it wide application prospect over Cu or Ag nanoparticles.

## Discussion

Novel Cu network supported nanoporous Ag-Cu alloy that having an Ag shell and an Ag-Cu core together with small nanopore of ~15 nm, large nanopore of ~40 nm and ligament of ~60 nm was synthesized by one-pot chemical dealloying of Zr-Cu-Ag-Al amorphous/crystalline composite. The formation of the Cu network supported nanoporous Ag-Cu@Ag core-shell alloy was confirmed to be closely related to the oxygen addition, which provides a new and simple way to prepare metallic core–shell nanostructures. The nanoporous Ag-Cu@Ag core-shell alloy exhibits excellent air-stability at room temperature and better oxidation resistance before 482 K, which is higher than that of reported core-shell Ag-Cu particles in microscale. In addition, the nanoporous Ag-Cu@Ag core-shell alloy also shows robust antibacterial activity against *E. Coli* DH5α. The simple and low-cost preparation method as well as the excellent oxidative stability and antibacterial activity promise the Ag-Cu core-shell nanoporous alloy to be widely used.

Since many MGs with various compositions have been developed, through the chemical dealloying treatment of their amorphous/crystalline composite, different core-shell nanoporous alloys could be obtained under proper conditions. This work thus may provide a new way to prepare diverse core–shell nanostructured materials in a simple chemical dealloying process at low cost.

## Methods

### Zr_38_Cu_40_Ag_7_Al_7_O_8_ amorphous/crystalline composite ribbon and Zr_48_Cu_36_Ag_8_Al_8_ MG ribbon preparation and characterization

Zr_38_Cu_40_Ag_7_Al_7_O_8_ (at.%) ingot were prepared by arc-melting the mixtures of pure Zr, Cu, Ag and Al (>99.4 mass%) in an argon atmosphere with small amount of air. (The true composition of the Zr-Cu-Ag-Al-O ingot was investigated by EDS in SEM.) To achieve chemical homogeneity, each ingot was remelted for at least 4 times. Then Zr-Cu-Ag-Al-O ribbons with dimensions of ~6 mm in width and ~70 μm in thickness were prepared by employing a single-roller melt-spinner. Zr_48_Cu_36_Ag_8_Al_8_ (at.%) MG ribbon was prepared by the same procedure but without air introducing during the arc-melting process. The Zr_48_Cu_36_Ag_8_Al_8_ MG ribbon possesses a wide of ~8 mm and a thickness of ~30 μm. The structure of the as-prepared Zr_38_Cu_40_Ag_7_Al_7_O_8_ and Zr_48_Cu_36_Ag_8_Al_8_ ribbon samples with dimensions of ~6 mm × 8 mm × 70 μm and ~8 mm × 8 mm × 30 μm, respectively, were examined by Rigaku D/max-RB XRD with monochromatic Cu Kα radiation. All the XRD spectra were obtained at a scanning rate of 8 degrees per min with a detecting step of 0.02 degree. All the EDS experiments were operated at a voltage of 20 KV with an acquisition time of ~5 min to make selected elements (Cu, Al, O) K shell peak over 10000 counts and (Zr, Ag) L shell peak over 5000 counts.

### Chemical dealloying procedure

Since the Zr-based metallic glasses can be easily etched by hydrofluoric acid, the hydrofluoric acid solution was chosen as the dealloying solution for the Zr-Cu-Ag-Al-O ribbon. The as-prepared ribbons were cut into pieces with ~4 cm in length and then dealloyed in 1 L hydrofluoric acid with concentration of 0.01 M, 0.05 M and 0.25 M under air atmosphere. After dealloying for different time, the dealloyed samples were removed from the solution, washed in ethanol, and then allowed to dry in air. All experiments were performed at room temperature.

### Dealloyed samples characterization

The structures of the dealloyed Zr_38_Cu_40_Ag_7_Al_7_O_8_ and Zr_48_Cu_36_Ag_8_Al_8_ samples with dimensions of ~6 mm × 8 mm × 30 μm and ~8 mm × 8 mm × 15 μm, respectively, were examined again by Rigaku D/max-RB XRD. The surfaces of the dealloyed samples were examined by LEO 1530 SEM at an operating voltage of 10 kV. The compositions of the as-prepared and dealloyed sample were investigated by EDS in SEM. The composition data were obtained by the statistics of more than 3 points on different areas of the samples. Then the nanoporous Ag-Cu@Ag core-shell alloy was dispersed in ethanol by ultrasound vibration and then collected by a copper grid with holey carbon film for TEM observing by a JEOL 2011 TEM operated at 200 kV. The TEM bright field image and dark field image were recorded by a CCD detector, and the diffraction patterns were recorded by films.

### Anti-oxygenic property testing of the Cu network supported nanoporous Ag-Cu@Ag core-shell alloy

The thermal stability of the nanoporous Ag-Cu@Ag core-shell alloy that dealloyed in 0.05 M hydrofluoric acid was investigated by Netzsch STA 449F3 DSC and TGA instrument at a heating rate of 10 K min^−1^ in air. After oxidized at different temperatures in air, the structures of the nanoporous Ag-Cu@Ag core-shell alloy with diameters of ~6 mm × 5 mm × 30 μm were investigated by Rigaku D/max-RB XRD.

### Antibacterial activity testing of the Cu network supported nanoporous Ag-Cu@Ag core-shell alloy

The antibacterial investigations on the nanoporous Ag-Cu@Ag core-shell alloy that dealloyed in 0.05 M hydrofluoric acid were performed against *Escherichia coli* DH5alpha strains. Luria-Bertani (LB) medium was used as a carrier to dilute the nanoporous Ag-Cu@Ag core-shell alloy solutions to different concentrations by ultrasonic. The monoclonal *E. coli* DH5α cells were grown over-night in 20 ml LB medium at 310 K on a Shaker (BHWY−100, Safe, Zhejiang, China), and then diluted to OD of 0.11 and 0.03 (at 600 nm) with volumes of 20 ml by different nanoporous Ag-Cu@Ag core-shell alloy solution. Also the controls were prepared under the same conditions, *i.e*., bacteria, LB medium and water, but without supplements of nanoporous Ag-Cu@Ag core-shell alloy. Then the *E. coli* DH5α cells were cultured at 310 K on the Shaker for 18 h. The change in the optical density at the wavelength of 600 nm was monitored by using a UV-vis spectrophotometer (Unico 2800, Unico, Shanghai, China) with a interval of every 2 h.

### Data availability

All data generated or analysed during this study are included in this published article (and its Supplementary Information files).

## Electronic supplementary material

Supporting Information: One-pot preparation of nanoporous Ag-Cu@Ag core-shell alloy with enhanced oxidative stability and robust antibacterial activity

## References

[1] Ge XB (2013). A Core-Shell Nanoporous Pt-Cu Catalyst with Tunable Composition and High Catalytic Activity. Adv. Funct. Mater..

[2] Nasir ME, Dickson W, Wurtz GA, Wardley WP, Zayats AV (2014). Hydrogen Detected by the Naked Eye: Optical Hydrogen Gas Sensors Based on Core/Shell Plasmonic Nanorod Metamaterials. Adv. Mater..

[3] Liu X, Shao Y, Tang Y, Yao KF (2014). Highly Uniform and Reproducible Surface Enhanced Raman Scattering on Air-stable Metallic Glassy Nanowire Array. Sci. Rep..

[4] Liu X, Zhao SF, Shao Y, Yao KF (2014). Facile synthesis of air-stable nano/submicro dendritic copper structures and their anti-oxidation properties. RSC Adv..

[5] Xiong B (2013). Highly sensitive sulphide mapping in live cells by kinetic spectral analysis of single Au-Ag core-shell nanoparticles. Nat. Commun..

[6] Tchaplyguine M, Andersson T, Zhang C, Bjorneholm O (2013). Core-shell structure disclosed in self-assembled Cu-Ag nanoalloy particles. J. Chem. Phys..

[7] Tsai CH, Chen SY, Song JM, Chen IG, Lee HY (2013). Thermal stability of Cu@Ag core-shell nanoparticles. Corros. Sci..

[8] Garcia S (2013). Microwave synthesis of Au-Rh core-shell nanoparticles and implications of the shell thickness in hydrogenation catalysis. Chem. Commun..

[9] Yoshida Y, Uto K, Hattori M, Tsuji M (2014). Synthesis and growth mechanism of Au@Cu core-shell nanorods having excellent antioxidative properties. Crystengcomm.

[10] Radmilovic V (2011). Highly monodisperse core-shell particles created by solid-state reactions. Nat. Mater..

[11] Hsieh YC (2013). Ordered bilayer ruthenium-platinum core-shell nanoparticles as carbon monoxide-tolerant fuel cell catalysts. Nat. Commun..

[12] Wang DL (2013). Structurally ordered intermetallic platinum-cobalt core-shell nanoparticles with enhanced activity and stability as oxygen reduction electrocatalysts. Nat. Mater..

[13] Chung RJ, Shih HT (2014). Preparation of Multifunctional Fe@Au Core-shell Nanoparticles with Surface Grafting as a Potential Treatment for Magnetic Hyperthermia. Materials.

[14] Mottaghi N (2014). Ag/Pd core-shell nanoparticles by a successive method: Pulsed laser ablation of Ag in water and reduction reaction of PdCl2. Appl. Surf. Sci..

[15] Shin J, Lee KY, Yeo T, Choi W (2016). Facile One-pot Transformation of Iron Oxides from Fe2O3 Nanoparticles to Nanostructured Fe3O4@C Core-Shell Composites via Combustion Waves. Sci. Rep..

[16] Yao QL, Lu ZH, Zhang ZJ, Chen XS, Lan YQ (2014). One-pot synthesis of core-shell Cu@SiO2 nanospheres and their catalysis for hydrolytic dehydrogenation of ammonia borane and hydrazine borane. Sci. Rep..

[17] Taner M, Sayar N, Yulug IG, Suzer S (2011). Synthesis, characterization and antibacterial investigation of silver-copper nanoalloys. J. Mater. Chem..

[18] Valodkar M, Modi S, Pal A, Thakore S (2011). Synthesis and anti-bacterial activity of Cu, Ag and Cu-Ag alloy nanoparticles: A green approach. Mater. Res. Bull..

[19] Hai HT, Takamura H, Koike J (2013). Oxidation behavior of Cu-Ag core-shell particles for solar cell applications. J. Alloy Compd..

[20] Jaiswal S, McHale P, Duffy B (2012). Preparation and rapid analysis of antibacterial silver, copper and zinc doped sot-gel surfaces. Colloid Surf. B-Biointerfaces.

[21] Zhang QS, Zhang W, Inoue A (2006). New Cu-Zr-based bulk metallic glasses with large diameters of up to 1.5 cm. Scripta Mater..

[22] Gong P (2013). A Ti-Zr-Be-Fe-Cu bulk metallic glass with superior glass-forming ability and high specific strength. Intermetallics.

[23] Liu X, Chen N, Gu JL, Du J, Yao KF (2015). Novel Cu-Ag bimetallic porous nanomembrane prepared from a multi-component metallic glass. RSC Adv..

[24] Erlebacher J, Aziz MJ, Karma A, Dimitrov N, Sieradzki K (2001). Evolution of nanoporosity in dealloying. Nature.

[25] Abe H (2009). Dealloying of Cu-Zr-Ti Bulk Metallic Glass in Hydrofluoric Acid Solution. Mater. Trans..

