# Supplementary material for: One-pot preparation of nanoporous Ag-Cu@Ag core-shell alloy with enhanced oxidative stability and robust antibacterial activity
Source: Sci Rep. 2017 Aug 31;7:10249. doi: 10.1038/s41598-017-10630-5 (PMC5579282; doi:10.1038/s41598-017-10630-5)
Supplement: Supplementary file 1 — Supporting Information: One-pot preparation of nanoporous Ag-Cu@Ag core-shell alloy with enhanced oxidative stability and robust antibacterial activity [file 41598_2017_10630_MOESM1_ESM.doc]

Supporting Information

One-pot preparation of nanoporous Ag-Cu@Ag core-shell alloy with enhanced oxidative stability and robust antibacterial activity

Xue Liu^1,2^, Jing Du^3^, Yang Shao^1^, Shao-Fan Zhao^1,4^ and Ke-Fu Yao^1^*

*^1^School of Materials Science and Engineering, Tsinghua University, Beijing 100084, People’s Republic of China*

*^2^Institute of Materials, China Academy of Engineering Physics, Mianyang 621900, People’s Republic of China*

*^3^Institute of Biomechanics and Medical Engineering, School of Aerospace, Tsinghua University, Beijing 100084, People’s Republic of China*

*^4^Qian Xuesen Laboratory of Space Technology, Beijing 100094, People’s Republic of China*

Fax: +86 10 6277 1160; Tel: +86 10 6277 2292; E-mail: kfyao@tsinghua.edu.cn


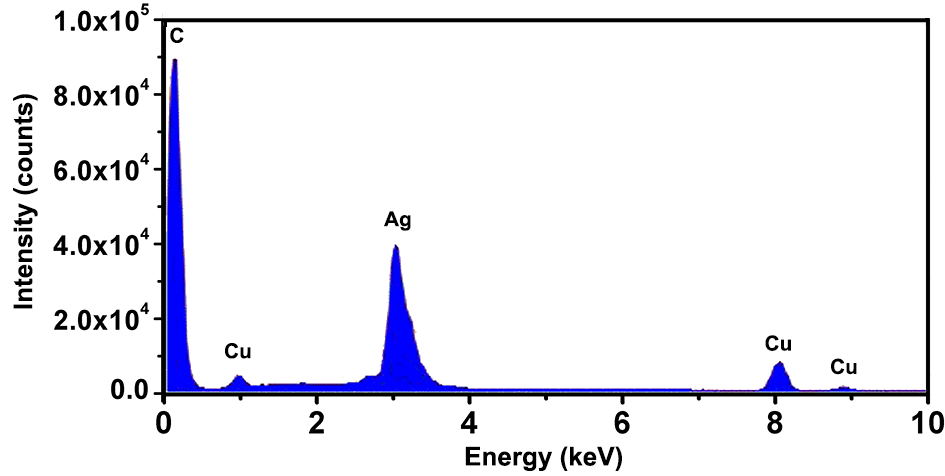


**Figure S1.** The energy dispersive spectrometer (EDS) spectrum of the nanoporous Ag-Cu@Ag core-shell alloy.


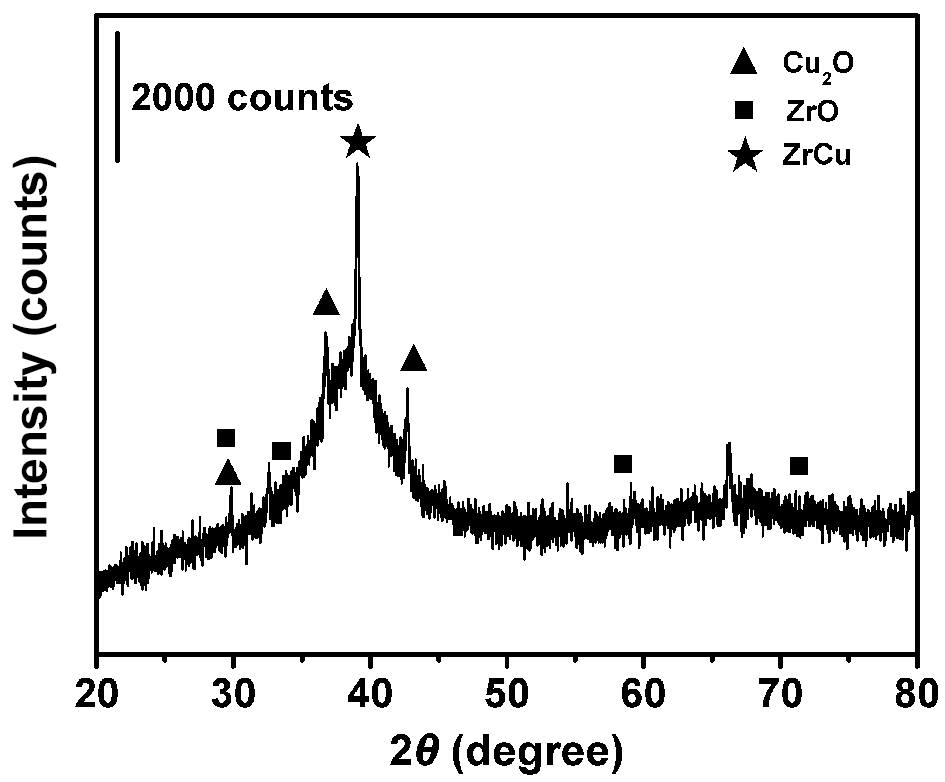


**Figure S2.** The X-ray diffraction (XRD) spectra of the as-prepared Zr_38_Cu_41_Ag_8_Al_7_O_7_ ribbon sample.


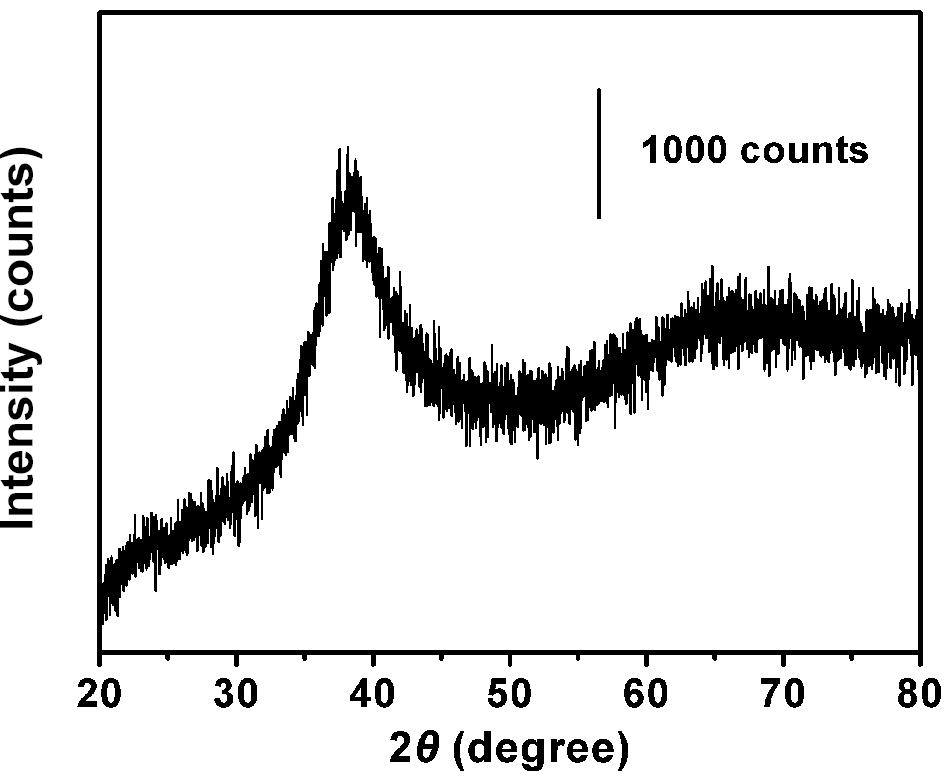


**Figure S3.** The XRD spectra of the as-prepared Zr_48_Cu_36_Ag_8_Al_8_ ribbon sample.


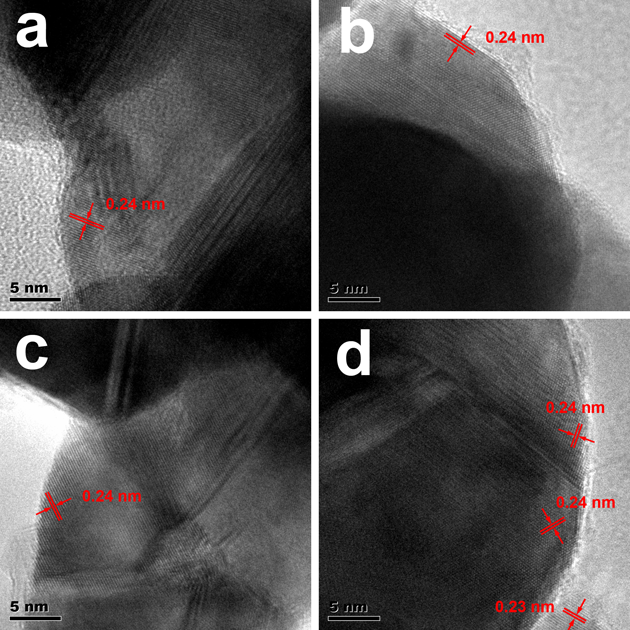


**Figure S4.** The high-resolution transmission electron microscopy (HRTEM) images of the prepared nanoporous Ag-Cu@Ag core-shell alloy.


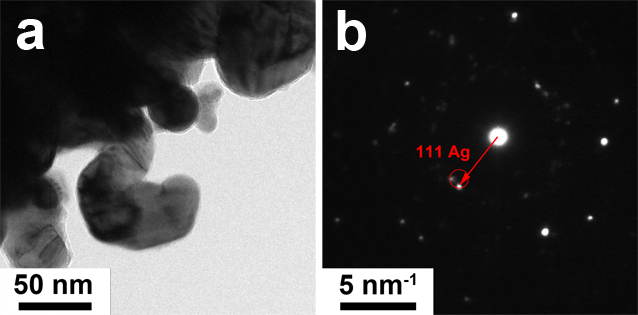


**Figure S5.** (a) The transmission electron microscopy (TEM) bright field image of the prepared nanoporous Ag-Cu@Ag core-shell alloy and (b) its corresponding diffraction pattern.


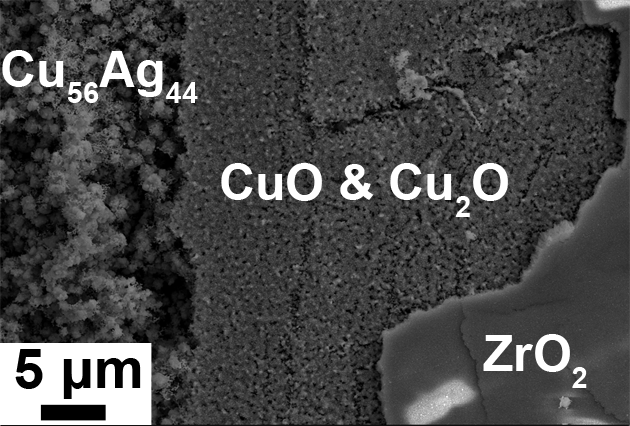


**Figure S6.** The scanning electron microscope (SEM) image of the Zr_48_Cu_36_Ag_8_Al_8_ MG ribbon that was subjected to oxidation in the air at 473 K for 30 min and then dealloyed under the same condition as the Zr-Cu-Ag-Al-O ribbon.

**
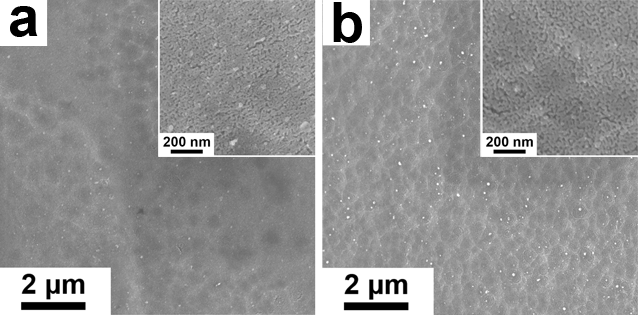
**

**Figure S7.** SEM images of two typical samples dealloyed for (a) 10 min and (b) 1 h.


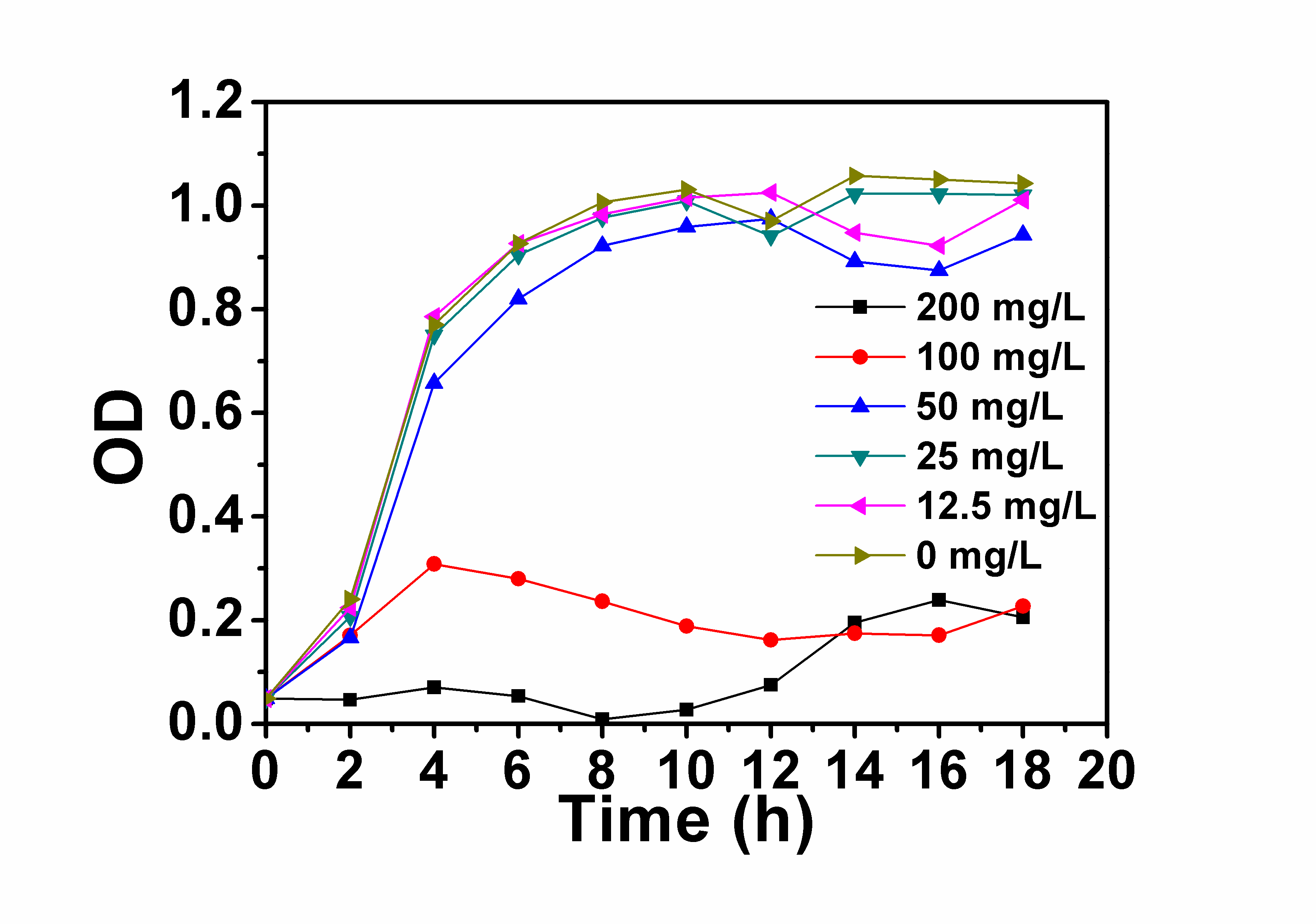

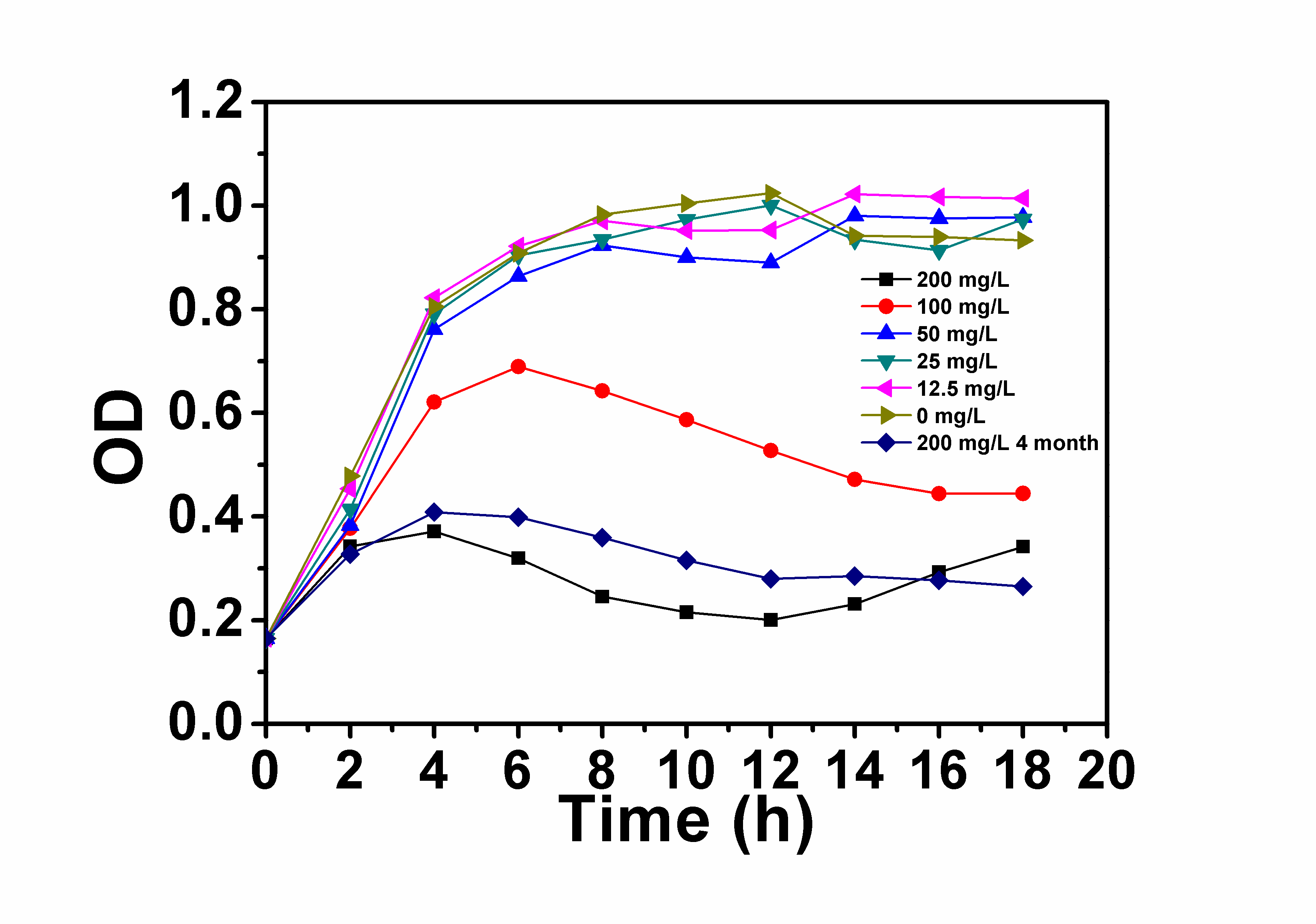


**Figure S8.** Antibacterial activity of the nanoporous Ag-Cu@Ag core-shell alloy. Representative batch growth profiles in the presence of nanoporous Ag-Cu@Ag core-shell alloy for initial concentrations of *E. Coli* DH5α: (a) 0.05 OD and (b) 0.16 OD.

**Table S1. Compositions of the samples dealloyed for different time.**

| Dealloying time | Compositions (at.%) | | | | |
| --- | --- | --- | --- | --- | --- |
|  | Zr | Cu | Ag | Al | O |
| 0 min | 37.9±0.7 | 40.3±1.1 | 7.4±0.1 | 6.6±0.5 | 7.8±0.6 |
| 10 min | 15.9±0.4 | 46.5±0.8 | 8.9±0.1 | 1.9±0.1 | 26.9±0.6 |
| 1 h | 1.1±0.7 | 76.6±3.2 | 14.2±0.5 | 1.1±0.7 | 8.1±2.3 |
| 24 h | 0 | 43.9±1.5 | 56.1±1.5 | 0 | 0 |
